# α‐Methoxy Benzaldehyde Based Photopolymers as a Promising Toolbox for Architected Carbon

**DOI:** 10.1002/marc.202500175

**Published:** 2025-03-25

**Authors:** Kjetil Baglo, Laurent Remy, Kai Mundsinger, Jan Torgersen, Christopher Barner‐Kowollik

**Affiliations:** ^1^ Chair of Materials Science, Department of Materials Engineering School of Engineering and Design Technical University of Munich (TUM) Boltzmannstr. 15 85748 Garching Germany; ^2^ School of Chemistry and Physics Centre for Materials Science Queensland University of Technology (QUT) 2 George Street Brisbane QLD 4000 Australia; ^3^ Institute of Nanotechnology (INT) Karlsruhe Institute of Technology(KIT) Hermann‐von‐Helmholtz‐Platz 1 76344 Eggenstein‐Leopoldshafen Germany; ^4^ Institute of Functional Interfaces (IFG) Karlsruhe Institute of Technology (KIT) Hermann‐von‐Helmholtz‐Platz 1 76344 Eggenstein‐Leopoldshafen Germany

**Keywords:** architected carbon, microspheres, photo Diels–Alder chemistry, pyrolysis

## Abstract

Stable carbonized microspheres can readily be obtained with highly efficient char yields of close to 60% from photopolymers based on photo‐induced Diels–Alder step growth polymerizations of α‐methoxy benzaldehyde and bismaleimide precursors. The current study carefully elucidates the chemical decomposition pathways during the pyrolysis of the microspheres that yield excellent char yields via thermogravimetric analysis (TGA) and infrared (IR) spectroscopy. The high char yield and low shrinkage of close to 33% make Diels–Alder‐type photopolymers a promising system for the next generation of additively manufactured carbon (AMcarbon) precursors.

## Graphical abstract

The photo‐induced Diels–Alder step‐growth polymerization of difunctional α‐methoxy benzaldehyde and bismaleimide precursors affords photopolymers shaped into microspheres that are ideally suited for the formation of tailored carbon geometries. Stable carbonized microspheres with close to 60% char yield and 33% shrinkage can be readily formed at a temperature of 1200 °C.

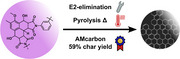

## 1 Introduction

Additively manufactured carbon (AMcarbon) – also known as architected carbon – is a promising material for microfluidic and electrochemical devices.^[^ ^1^, ^2^, ^3^, ^4^, ^5^, ^6^, ^7^, ^8^ ^]^ Such devices often require freedom in geometry to accommodate challenging transport kinetics, where AMcarbon offers tailored structural features (i.e., beam diameter, thickness, geometry, porosity, pore size, porosity distribution, and surface morphology^[^ ^9^, ^10^ ^]^). The material is generated through pyrolysis of a photopolymer precursor, while simultaneously retaining its relative dimensions and predefined 3D shape. During the pyrolysis step, the photopolymer shrinks and loses mass that does not necessarily result in solid carbon. The mass lost during pyrolysis is transported through the polymeric material, causing pores and cracks to form as well as distortion of the part's shape leading to warping.^[^ ^11^, ^12^, ^13^ ^]^ Conventional and state‐of‐the‐art photopolymers shrink significantly (>50% in dimensions) and lose the majority of their mass (>80 wt.%), making the fabrication of larger parts challenging.^[^ ^11^, ^12^, ^13^ ^]^ The lack of development of suitable photopolymeric precursors has recently been pointed out, with most authors focusing on the application rather than the material.^[^ ^14^ ^]^

It appears that some of the challenges during the pyrolysis process arise from low mass retention and high shrinkage. An ideal starting point for material development is identifying photopolymers that retain more of their mass during pyrolysis. There is extensive literature on what endows an organic precursor with a high residual mass, also known as char yield (i.e., the weight fraction of retained carbon atoms after pyrolysis).^[^ ^15^ ^]^ A high char yield precursor should possess the following properties: formation of cyclic intermediates, ring fusion, chain coalescence, free radical formation upon C─H or C─C bond cleavage, and high aromatic content with no more than two aliphatic carbons between aromatic units.^[^ ^16^, ^17^, ^18^, ^19^, ^20^ ^]^ The majority of photopolymers for vat photopolymerization utilize acrylic, methacrylic, and epoxy functional groups that do not form polymers with these properties, typically resulting in char yields below 20%.^[^ ^7^, ^12^, ^15^, ^16^, ^21^, ^22^, ^23^ ^]^

Such char yields are particularly low compared to the long‐standing polymeric carbon precursors such as phenol formaldehyde polymers, featuring char yields between 62% and 68%.^[^ ^24^ ^]^ Unfortunately, phenol formaldehyde is not photopolymerizable and the need for development of photopolymers with high char yield has recently been raised.^[^ ^14^ ^]^ α‐methoxy benzaldehyde‐based photopolymers are a family of polymers that exhibit many of the desired traits of a precursor and have recently been demonstrated to be efficient precursors for the fabrication of microspheres.^[^ ^25^ ^]^ Herein, we demonstrate how α‐methoxy benzaldehyde and bismaleimide copolymers improve char yields and lower shrinkage by carbonizing microspheres based on these photopolymers as a model system.

## 2 Results and Discussion

### 2.1 Light‐Induced Synthesis of Polymer Microspheres and Hydroxyl Elimination

**Scheme 1:**
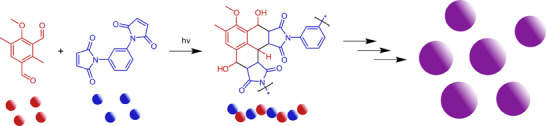
Synthetic route for polymeric microspheres via a Diels–Alder step‐growth polymerization. For details of the microsphere formation process refer to.^[^ ^25^ ^]^

In our previous studies,^[^ ^25^ ^]^ difunctional reactive intermediate photoenols derived from methylisophtalaldehyde precursors were found to be versatile photoactive intermediates for particle synthesis in the presence of difunctional maleimide precursors via light‐induced step‐growth polymerization. During irradiation, the photoactive monomer sequentially generates two highly reactive *ortho*‐quinodimethane (*o*‐QDM) intermediates. These intermediates can subsequently undergo a Diels–Alder cycloaddition reaction with the diene moieties of the bismaleimide monomer. The resulting oligomers grow until reaching a critical molar mass, at which phase separation occurs, and the resulting precipitates form nuclei that continue to grow, yielding stable polymeric particles (**Scheme** **1**).^[^ ^25^ ^]^

**Scheme 2:**
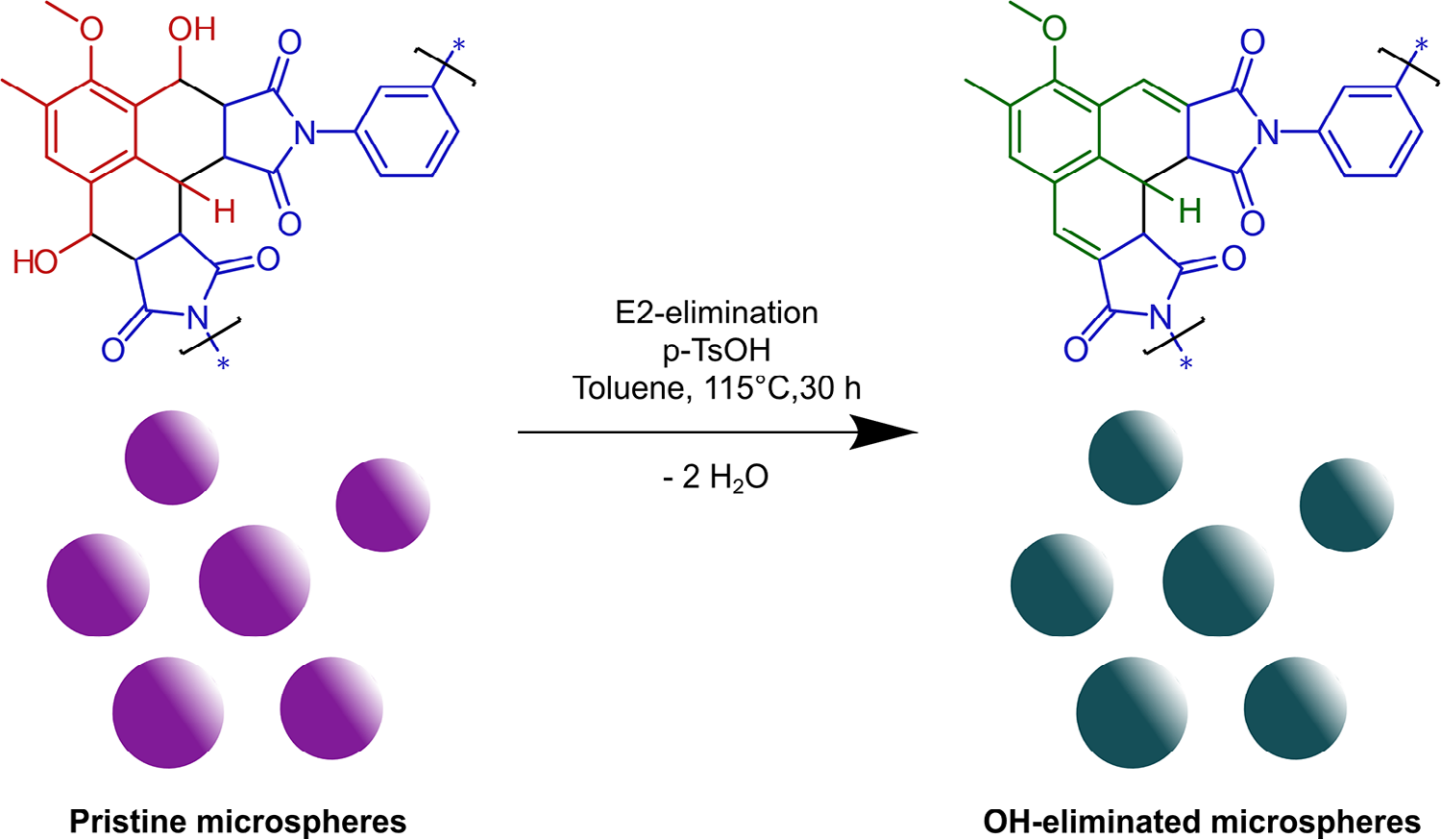
Hydroxyl elimination from the Diels–Alder adduct as described by us previously.^[^ ^27^ ^]^

Following our exploratory study,^[^ ^25^ ^]^ which laid the foundations of crosslinker free Diels–Alder based formation of well‐defined microparticles, we investigated novel dialdehyde structures and synthetic methods,^[^ ^26^ ^]^ as well as post‐modification of the obtained microparticles.^[^ ^27^ ^]^ In the present study, we explore 4‐methoxy‐2,5‐dimethylisophthalaldehyde and N,N’‐(1,3‐phenylene)dimaleimide as monomers for photochemical microsphere synthesis.^[^ ^28^ ^]^ Expectedly, after 8 h of LED irradiation centered at 365 nm, polymer microspheres were obtained (Figure S15, Supporting Information) and consecutively purified via repeated centrifugation and dispersing in organic solvents.^[^ ^25^ ^]^ The resulting microspheres were subsequently treated with *para*‐toluenesulfonic acid monohydrate in toluene resulting in E2‐elimination (referred to as OH‐elimination) of the secondary hydroxyl groups generated during the synthesis, resulting in a phenalene‐type motif (**Scheme** **2**). The completion of the OH‐elimination has been demonstrated both for polymer microspheres and small molecules based on the same photochemistry.^[^ ^27^, ^28^ ^]^ Details of the synthesis of the precursor monomers, the microspheres, and the OH‐elimination can be found in procedures S1–S4 (Supporting Information).

### 2.2 Microsphere Pyrolysis

We employed TGA and FT‐IR spectroscopy to map the pyrolysis profiles of both pristine and OH‐eliminated microspheres to establish the differences in their chemical degradation mechanism and elucidate why the examined photopolymer system is an ideal precursor material for pyrolysis with high char yields.

**Figure 1:**
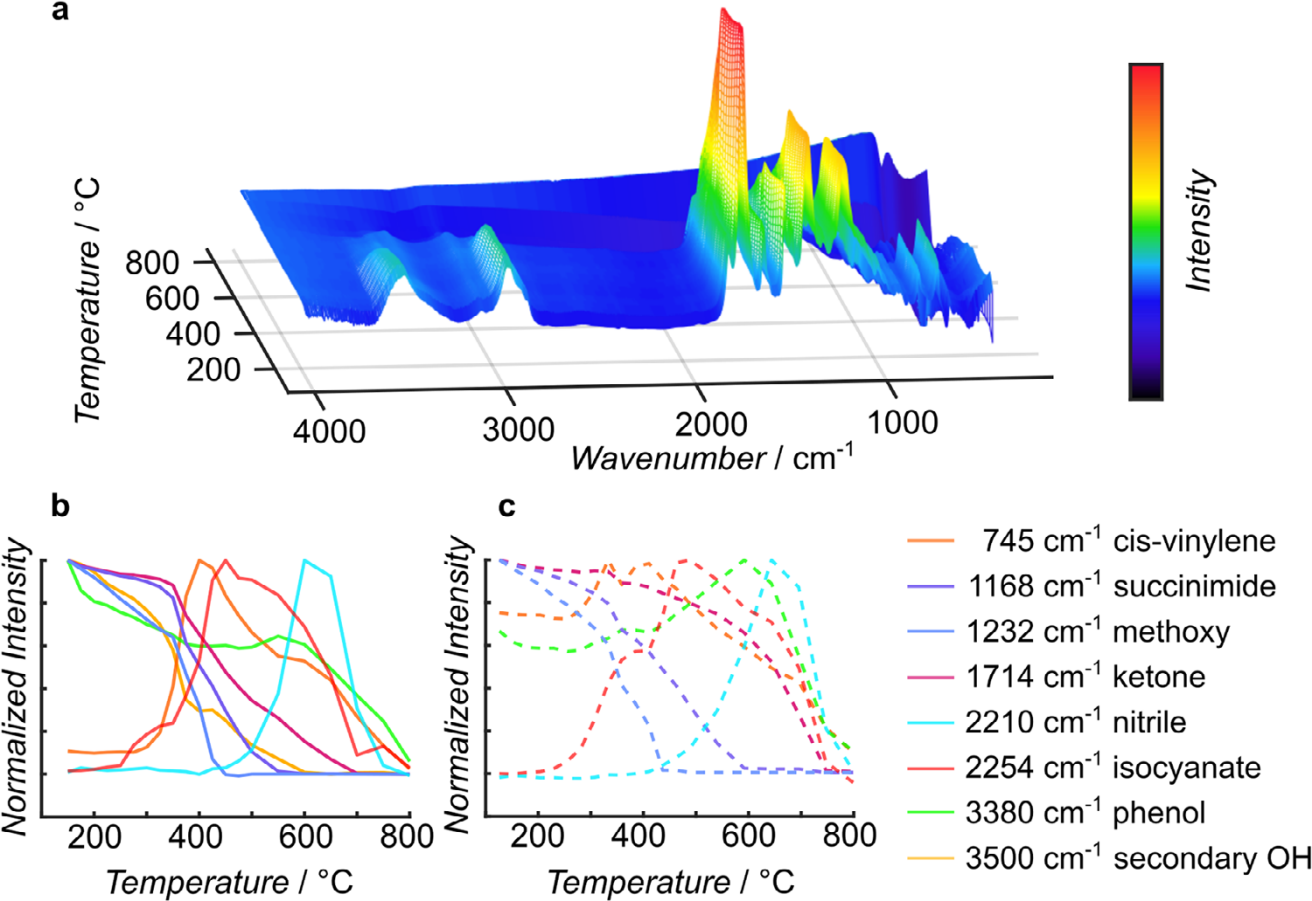
a) 3D‐Plot of the emission FTIR spectra of the pristine α‐methoxy benzaldehyde based photopolymer from 200 to 800 °C; b) local intensity plotted versus temperature for the pristine microspheres (solid lines) to monitor the formation and consumption of selected motifs; c) local intensity plotted versus temperature for the OH‐eliminated microspheres (dashed lines). 2D stacked plots of the recorded FTIR spectra are given in Figures S3–S6 (Supporting Information) for the pristine microspheres and Figures S7–S10 (Supporting Information) for the OH‐eliminated microspheres. Figure S11 (Supporting Information) displays an overlay of graphs (b,c) for ease of comparison.

To establish the thermal behavior of the microspheres, changes in their chemical structure were analyzed between 150 to 800 °C via emission FTIR spectroscopy (**Figure** **1**). All recorded spectra are available as stacked 2D plots in Figures S3–S10 (Supporting Information), and plotted as a 3D graph for the pristine microspheres in Figure 1a. The local intensity, measured as the peak intensity for a selected vibrational mode compared to a linear background, is evaluated for selected molecular vibrations as illustrated in Figure S12 (Supporting Information). This local intensity is normalized and plotted versus the temperature to establish the temperature‐dependent formation and consumption of the chemical transformation associated with the selected vibrations (refer to Figure 1b for the non‐modified and Figure 1c for the OH‐eliminated microspheres). The initial changes to the pristine spheres as temperature increases are observed for the hydroxyl (─OH) vibrations at 1068 and 3500 cm^−1^. These are attributed to the secondary alcohol formed during the Diels–Alder cycloaddition. These start to diminish in intensity at 300 °C and are no longer observable at 425 °C. Simultaneously, the 745 cm^−1^ *cis*‐vinylene vibration emerges, increases in intensity until 400 °C, and subsequently starts to diminish before complete consumption at 700 °C. The OH‐eliminated microspheres do not feature the vibrations at 1068 and 3500 cm^−1^ and exhibit the *cis*‐vinylene vibration at 745 cm^−1^ at all temperatures up to 400 °C as expected from structures where the hydroxyl moieties were previously eliminated to yield *cis*‐vinylene motifs.

Both pristine and OH‐eliminated microspheres show the formation of a phenol‐type motif, indicated by the broad vibration at 3380 cm^−1^ in the FTIR spectra. This vibration becomes prominent at 375 °C and persists up to 700 °C. There is a considerable overlap between the aromatic and secondary hydroxyl vibrations and care must be taken when evaluating the local intensities. The C═O vibration appears at 1714 cm^−1^ and starts to decrease in intensity at 375 °C for the pristine microspheres before complete consumption at 700 °C, whereas the C═O vibration for the OH‐eliminated micro spheres starts to diminish appreciably at 500 °C and is fully consumed at 750 °C. The vibrations at 684 and 1168 cm^−1^ are caused by the succinimide group, which starts to diminish at 375 °C and is completely consumed at 550 °C for both the pristine and OH‐eliminated spheres. The onset of this degradation mechanism is accompanied by the presence of a peak at 2254 cm^−1^ attributed to isocyanate groups, reaching a maximum at 425 to 450 °C, before consumption at 700 °C. The isocyanate vibration is much more pronounced for the OH‐eliminated spheres, 3.5 times that of the pristine microspheres. At 475 °C a peak at 2210 cm^−1^ is observed, persisting up to 750 °C. This vibration is associated with nitrile groups and has its highest intensity at 600 to 650 °C and is close to 2.8 times more prominent for the OH‐eliminated microspheres. Methoxy groups, associated with the COC stretching vibration at 1232 cm^−1^, start to degrade considerably at 375 °C and are consumed at 450 °C. Above 550 °C, the most prominent vibrations are at 3380, 3044, 2210,1714, 1568, 1368, 872, and 745 cm^−1^, which are all vanishing at 800 °C. The 745 cm^−1^ band is known to be associated with *cis‐*vinylene, the 1368 cm^−1^ band is likely to be associated with a methyl or methylene function, the 2210 cm^−1^ band is the nitrile vibration, while the 3044 cm^−1^ peak is associated with an aromatic hydrogen stretching vibration.^[^ ^29^ ^]^ The 1714 cm^−1^ band is associated with a ketone stretching vibration. This leaves the 872 and 1568 cm^−1^ vibrations, which are in the range expected for phenalene with vibrations at 754, 839, and 1546 cm^−1^ in an Ar matrix at 8–10 K.^[^ ^30^ ^]^

**Figure 2:**
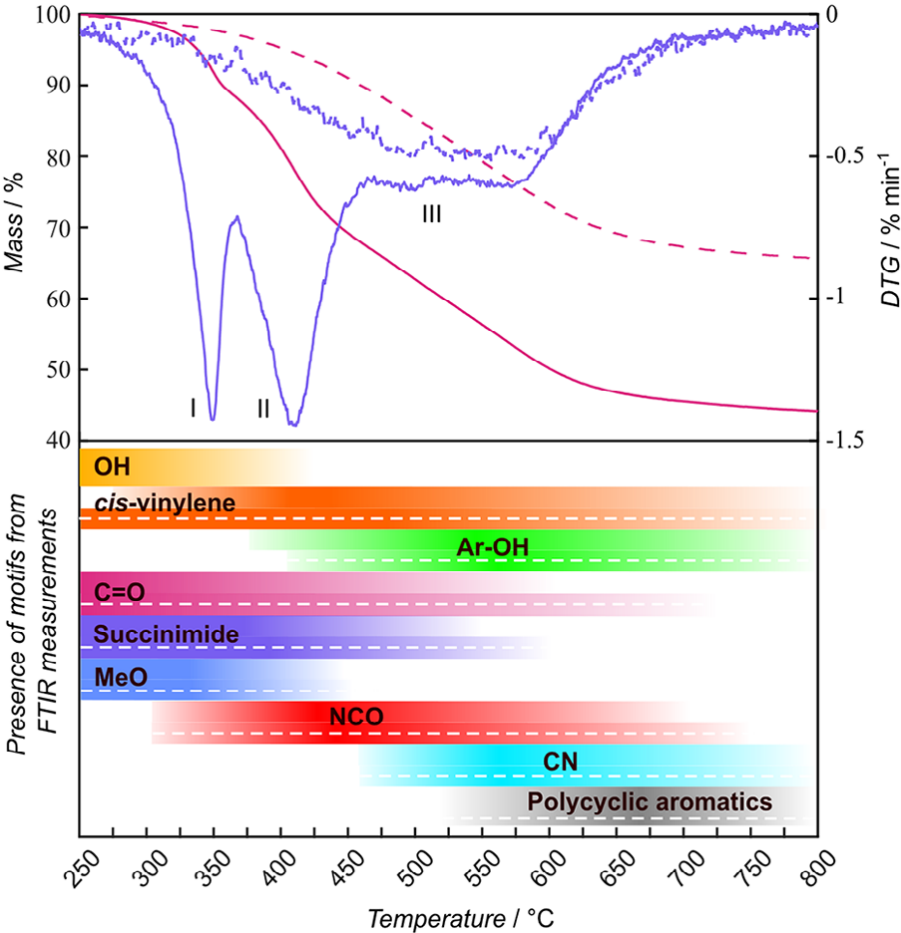
Thermogram and differential thermogram (DTG) of pristine (solid line) and OH‐eliminated (dashed line) microspheres displaying three weight loss ranges denoted I, II, and III. The presence of selected motifs as measured by FTIR are given below the thermogram. The thermogram is corrected for mass loss and cropped to display the relevant range investigated. The TGA for the full thermal range (30–1200 °C) can be found in Figure S13 (Supporting Information).

**Scheme 3:**
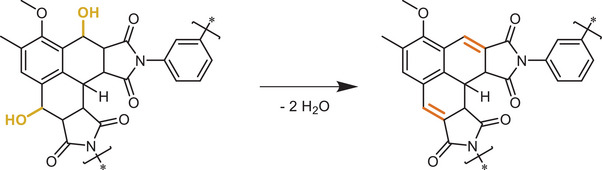
Hydroxyl elimination reaction was responsible for the initial mass loss recorded from 274 to 367 °C (Figure 2I). This elimination is analogous to the OH‐elimination using *para*‐TsOH.

The thermogram of the pristine and OH‐eliminated microspheres from 250 to 800 °C is depicted in **Figure** **2**, the char yield at 800 °C is close to 44% and at 1200 °C 41% for the pristine and 66% at 800 °C and 59% at 1200 °C for the OH‐eliminated microspheres. Such char yields are significantly higher than the previously highest reported char yield in the field of AMcarbon/architected carbon.^[^ ^21^ ^]^ The herein examined photopolymer based microspheres approach char yields of the benchmark system phenol formaldehyde (62–68%).^[^ ^21^, ^24^ ^]^ The onset of mass loss is estimated at 274 °C for the OH‐eliminated microspheres. The initial mass loss (I) reaches a maximum at 349 °C and depletes until exceeded by the second mass loss reaction at 367 °C, resulting in a mass loss of 12% at 367 °C, which is not observed for the OH‐eliminated microspheres. Based on the FTIR data, mass loss I is caused by the elimination of hydroxyl groups resulting in the formation of *cis*‐vinylene and water as shown in **Scheme** **3**. The theoretical mass loss from such a reaction based on molecular weight is 8%, which is 4% lower than the measured mass loss. Such a divergence is not unsurprising, considering the overlap with the subsequent reaction for the pristine microspheres.

**Scheme 4:**
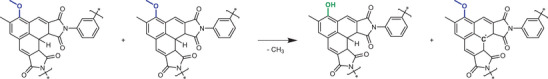
Decomposition of the methoxy group and subsequent hydrogen abstraction of tertiary carbon progressing between 375 and 600 °C, II, and III in the DTG displayed in Figure 2.

The second mass loss has its maximum at 410 °C (II) and appears to deplete at 475 °C for the pristine microspheres. Both the methoxy group and the succinimide ring are consumed in this temperature range. Degradation of the methoxy group is expected to follow the same pathway as is known for anisole, resulting in dissociation of methyl from the methoxy group.^[^ ^31^ ^]^ Such a reaction introduces radicals in the system facilitating hydrogen abstraction, and the most favorable carbon‐hydrogen bonds to dissociate are located at the tertiary carbons (simplified reaction in **Scheme** **4**).^[^ ^19^ ^]^ Other degradation pathways may occur, but the exact mechanisms leading to the detailed atom‐by‐atom degradation pathway are likely more complex and would require further analysis.

**Scheme 5:**
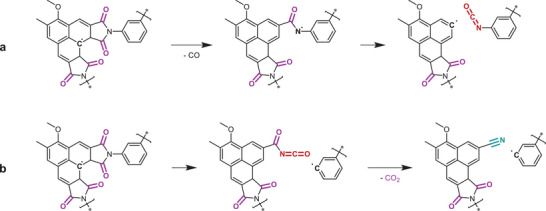
The pyrolysis path as measured by FTIR occurring from 375 to −650 °C, II and III of the DTG in Figure 2. a) isocyanate formation as reported for neat polymaleimides; b) isocyanate and nitrile formation reported for succinimide conjugated with aromatic motifs.

If dissociation of the methoxy group is assumed to be the dominating reaction in the 367 to 475 °C range (II), the expected residual mass upon completion would be 86%, much larger than the experimentally observed 66% at 475 °C for the pristine microspheres. It is likely that the majority of mass loss in this temperature range stems from the decomposition of the succinimide ring and aliphatic motifs resulting from incomplete hydroxyl elimination in tandem with Scheme 4. Mass loss II is not observed for the hydroxyl‐eliminated microspheres, likely due to a lower aliphatic content and resistance to fracture of the phenalene‐like motif. Aromatic maleimides are reported to degrade through the expulsion of carbon monoxide and the formation of isocyanates in an inert atmosphere, as illustrated in **Scheme** **5**a.^[^ ^32^ ^]^ The DTG appears constant (III) from 475 to 575 °C for both pristine and OH‐eliminated microspheres. In this range, nitrile functional groups were observed in the FTIR spectra at 2210 cm^−1^, reaching a maximum between 600 and 650 °C. To the best of our knowledge, nitrile moieties are not reported as typical degradation products of maleimides. However, for succinimide rings bonded with aromatic motifs, the formation of nitriles is reported, as drawn in Scheme 5b.^[^ ^33^ ^]^ The formation of the isocyanate functional group is observed from 375–650 °C, with its highest intensity at 425–450 °C, making it a likely product of the second and third mass loss reaction (II and III) observed in the thermogram. Both reactions a and b in Scheme 5 feature an isocyanate as either an intermediate or final product. For the OH‐eliminated microspheres, it is therefore not certain which reaction contributes most to (II). However, for neat polymaleimides, it is reported that the scission of the succinimide ring is more favorable than the cleavage of the N‐phenyl bond, making the reaction in Scheme 5a the more probable cause of the second mass loss reaction in combination with fracture of the phenalene like backbone.^[^ ^32^, ^34^ ^]^

At temperatures surpassing 550 °C, the most prominent groups discernible in the FTIR spectra are ketone, methylene/methyl, nitrile, hydroxyl substituted aromatics, and phenalenes, all congruent with the products depicted in Scheme 5. The subsequent conversion to a pure carbon allotrope is not discernible, yet is likely to progress through cleavage of non‐aromatic constituents and condensation of the aromatic backbones.^[^ ^20^ ^]^

### 2.3 Sphere Size and Shrinkage

**Figure 3:**
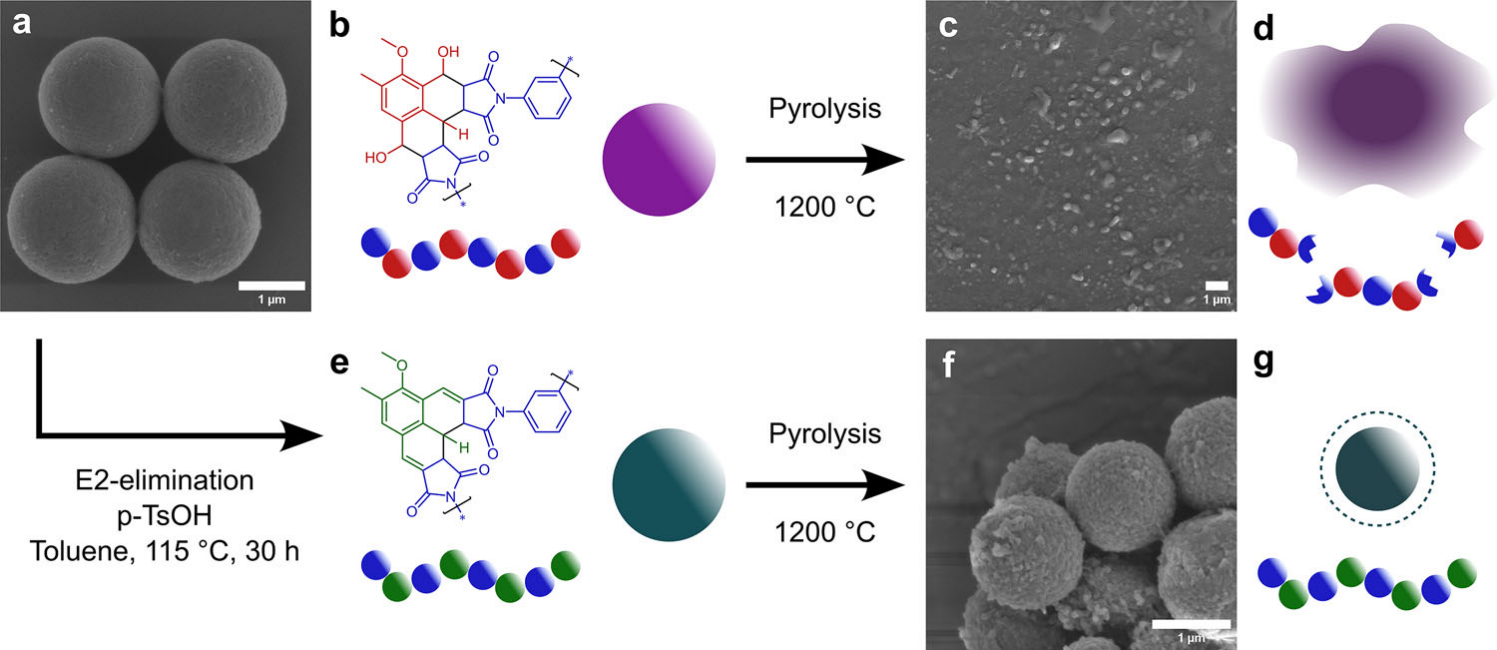
a) Synthesized microspheres with their molecular structure b) prior to pyrolysis up to 1200 °C at 5 °C min^−^^1^ resulting in c) molten and carbonized microspheres. Melting is likely due to cleavage of the polymer backbone d). After the elimination of the hydroxyl groups prior to pyrolysis e), the shape of the spheres is retained, and uniform shrinkage is observed after pyrolysis up to 1200 °C (f,g).

The polymer microspheres pre‐ and post‐pyrolysis at 1200 °C are depicted in **Figure** **3**. The untreated microspheres **a** with the molecular structure given in **b** melt when heated to 1200 °C at 5 °C min^−1^ due to the fracture of the polymer backbone (**c** and **d**). In sharp contrast, however, the OH‐eliminated microspheres with the molecular structure given in **e** retain their shape during pyrolysis, resulting in stable carbon spheres experiencing uniform shrinkage (**d** and **f**). The microspheres have an average initial diameter of 1.8 ± 0.2 µm as measured by SEM imaging, Figure S14 (Supporting Information). The carbonized OH‐eliminated microspheres have an average diameter of 1.2 ± 0.2 µm, Figure S15 (Supporting Information), resulting in a shrinkage of close to 33% in diameter. For comparison, commonly used SU‐8 micropillars with thicknesses of 64–76.8 µm shrink by 52–60%, while affording char yields of 30% after pyrolysis at 1150 °C. Thus, our herein‐reported system clearly outperforms these commonly used architected carbon precursors.^[^ ^15^ ^]^

Although we demonstrated the potential of our Diels–Alder photopolymer as an AMcarbon precursor in terms of shrinkage and mass retention, there are remaining challenges. We highlight that microspheres are not optimal 3D structures for pyrolysis due to their high surface‐to‐volume ratio.^[^ ^15^ ^]^ The high char yield and low shrinkage merit further work on how to pattern, synthesize, and carbonize photoinitiated Diels–Alder cycloaddition polymers to enable free‐form geometries, which would greatly alleviate defect formation in AMcarbon. Translation of the presented photopolymer system into a 3D printable resin will potentially enable tailored carbon geometries with large defect‐free parts, which for example could in turn accelerate the research on electrodes for electrochemical devices.

## 3 Conclusion

We address oustanding challenges with current photopolymer‐derived pyrolysis processes, where a low char yield and high shrinkage during pyrolysis result in warping and kilning flaws in the final part. In contrast, herein, we demonstrate that polymeric microspheres based on a step growth photopolymerization of difunctional α‐methoxy benzaldehydes and bismaleimides can be readily carbonized with excellent char yields (i.e., percent of mass obtained after pyrolysis). A char yield close to 60% at 1200 °C and a shrinkage of approximately 33%, make our new system the best‐performing precursor for photopolymer‐derived carbon (AMcarbon) demonstrated to date.

## 4 Experimental Section

All materials and synthesis information can be found in the Supplementary Information section. 4‐hydroxy‐2,5‐dimethylisophthalaldehyde was synthesized by a Duff reaction from 2,5‐dimethylphenol (Procedure S1, Supporting Information) and methylated to yield 4‐methoxy‐2,5‐dimethylisophthalaldehyde (Procedure S2, Supporting Information), as described in a previous study.^[^ ^25^ ^]^

Flash chromatography was performed on an Interchim XS420+ flash chromatography system consisting of an SP‐in‐line filter 20‐µm, a UV–vis detector (200–800 nm), and a SofTA Model 400 ELSD (55 °C drift tube temperature, 25 °C spray chamber temperature, filter 5, EDR gain mode) connected via a flow splitter (Interchim Split ELSD F04590). The separations were performed using an Interchim dry load column (liquid injection) and an Interchim Puriflash Silica HP 30 µm column.

^1^H‐NMR spectra were recorded on a Bruker System 600 Ascend LH, equipped with a BBO‐Probe (5 mm) with z‐gradient (^1^H: 600.13 MHz). Resonances are reported in parts per million (ppm) relative to tetramethylsilane (TMS). The δ‐scale was calibrated to the respective solvent signal of CHCl_3_ on the middle signal of the CDCl_3_ triplet. The annotation of the signals was based on HSQC‐, COSY‐ and DEPT experiments.

**Figure 4:**
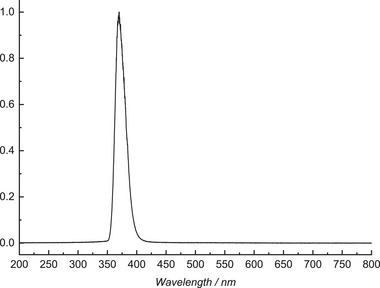
Emission spectra of the 365 nm 3W LED employed for microsphere formation.^[^ ^25^ ^]^

365 nm LED emission spectra were recorded using an Ocean Insight Flame‐T‐UV–vis spectrometer, with an active range of 200–850 nm and an integration time of 10 ms (**Figure** **4**). LED output energies were recorded using a Thorlabs S401C thermopile sensor, with an active area of 100 mm^2^ and a wavelength range of 190 nm–20 µm, connected to a Thorlabs PM400 energy meter console. The emitted power from each LED was measured for 60 s at a fixed distance from the sensor, after which the mean and standard deviation of the emission could be determined. LEDs were cooled during measurement to minimize any thermal effects on the emission power or sensor performance.

FTIR spectra are recorded on a Nicolet iS50 in emission mode with a custom furnace equipped with graphite and Pt emission sources. The spectra of the α‐methoxy benzaldehyde photopolymer microspheres are recorded on Pt ($\varepsilon_{S/\mathrm{Pt}}$) and corrected by measurements of the emission from the graphite (ideal black body $\varepsilon_{C}$) and from the Pt(background $\varepsilon_{\mathrm{Pt}}$), the correction is given in Equation (1).

(1) $$\varepsilon =\frac{\varepsilon_{S/\mathrm{Pt}}}{\varepsilon_{C}}\;-\frac{\varepsilon_{\mathrm{Pt}}}{\varepsilon_{C}}$$

Figures S3 and S7 (Supporting Information) display the background corrected and staked spectra at investigated temperatures.

Above 350 °C the intensity of the sample was too high and the MTC detector was saturated. To mitigate this physical limitation, a blinder (Nicolet screen A) was used at 375 °C and higher temperatures. The measurement settings used to record spectra are given in Table S1 (Supporting Information). Selected vibrations are subsequently evaluated for their local intensity by fitting a linear function to the peak regain and measuring the intensity of the peak from this fitted line, as illustrated in Figure 1b. The evaluated intensities are then plotted versus temperature to investigate the conversion to carbon (Figure S11, Supporting Information).

Thermogravimetric measurements are conducted on a Netzsch STA 449 C Jupiter with a 90 µL alumina crucible. 6.05 mg of microspheres are heated from 30 to 1200 °C at 5 °C min^−^^1^ in Argon atmosphere, the resulting thermogram was given in Figure S13 (Supporting Information). The mass loss was then corrected for solvent evaporation before numerically differentiated and smoothed, to plot the differential thermogram DTG.

## Conflict of Interest

The authors declare no conflict of interest.

## Supporting information



Supporting Information

## Data Availability

The data that support the findings of this study are available in the supplementary material of this article.
