# Supplementary material for: α‐Methoxy Benzaldehyde Based Photopolymers as a Promising Toolbox for Architected Carbon
Source: Macromol Rapid Commun. 2025 Mar 25;46(23):2500175. doi: 10.1002/marc.202500175 (PMC12687701; doi:10.1002/marc.202500175)
Supplement: Supplementary file 1 — Supporting Information [file MARC-46-2500175-s001.pdf]

Supporting Information

α-Methoxy Benzaldehyde Based Photopolymers as a Promising Toolbox for Architected Carbon

Kjetil Baglo,[#] Laurent Remy,[#] Kai Mundsinger, Jan Torgersen, Christopher Barner-Kowollik**

Kjetil Baglo, Prof. Dr. Jan Torgersen
Chair of Materials Science, Technical University of Munich (TUM), 15 Boltzmannstr., 85748 Garching (Germany)
E-mail: jan.torgersen@tum.de

Dr. Laurent Remy, Dr. Kai Mundsinger, Prof. Dr. Christopher Barner-Kowollik
School of Chemistry and Physics, Centre for Materials Science, Queensland University of Technology (QUT), 2 George Street, Brisbane, QLD 4000 (Australia)
E-mail: christopher.barnerkowollik@qut.edu.au

Prof. C. Barner-Kowollik, Institute of Nanotechnology (INT), Karlsruhe Institute of Technology(KIT) Hermann-von-Helmholtz-Platz 1, 76344 Eggenstein-Leopoldshafen (Germany)

Prof. C. Barner-Kowollik, Institute for Functional Interfaces (IFG), Karlsruhe Institute of Technology (KIT), Hermann-von-Helmholtz-Platz 1, 76344 Eggenstein-Leopoldshafen (Germany)

[#]These authors contributed equally to the study

Contents

Materials 2
Synthetic procedure 4-methoxy-2,5-dimethylisophthalaldehyde 2
Microsphere synthesis and E2-elimination 4
FTIR spectra 5
Thermogravimetric measurements 16
SEM images of microspheres 18
References 19

Materials

All materials were reagent grade and used as received, unless stated otherwise: 2,5-Dimethylphenol (Sigma-Aldrich), hexamethylenetetramine (Sigma-Aldrich), hydrochloric acid (32%, Thermo-Fisher), acetonitrile (ACN, HPLC-grade, Thermo-Fisher), methyl iodide (Sigma-Aldrich), anhydrous potassium carbonate (ChemSupply Australia), anhydrous magnesium sulphate (Merck), trifluoroacetic acid (Thermo-Fisher), anhydrous ethyl acetate (Sigma-Aldrich), cyclohexane (Merck), chloroform-*d* ($CDCl_3$, 99.8%D, Cambridge Isotope Laboratories).

Synthetic procedure 4-methoxy-2,5-dimethylisophthalaldehyde

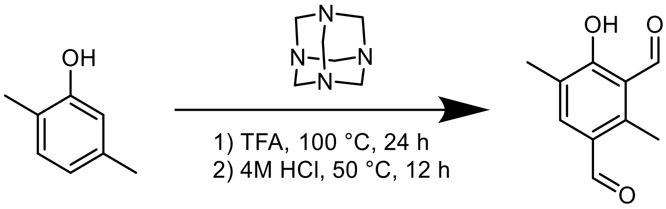

Procedure S1 – 4-hydroxy-2,5-dimethylisophthalaldehyde: The procedure is based on a previous study and utilized without further modification.[1] Briefly, 2,5-dimethylphenol (5.00 g, 40.93 mmol, 1.00 eq.) was dissolved in 32.7 mL of trifluoroacetic acetic acid (TFA) in a 250 mL round-bottom flask. Hexamethylenetetramine (20.80 g, 163.71 mmol, 4.00 eq) was subsequently added and the mixture stirred under argon at 100 °C for 24 h. After cooling to room temperature, 72 mL of 4 M HCl were added, and the media was heated to 50 °C under nitrogen flow for 12 h. The solution was allowed to cool to room temperature, diluted with 50 mL of water and left to cool in a refrigerator overnight. The resulting precipitate was filtered and washed with cold water and dried under vacuum. The crude product was purified by sublimation under reduced pressure at 60 °C, yielding the desired product as yellowish crystals (1.6195 g, 22 % yield).

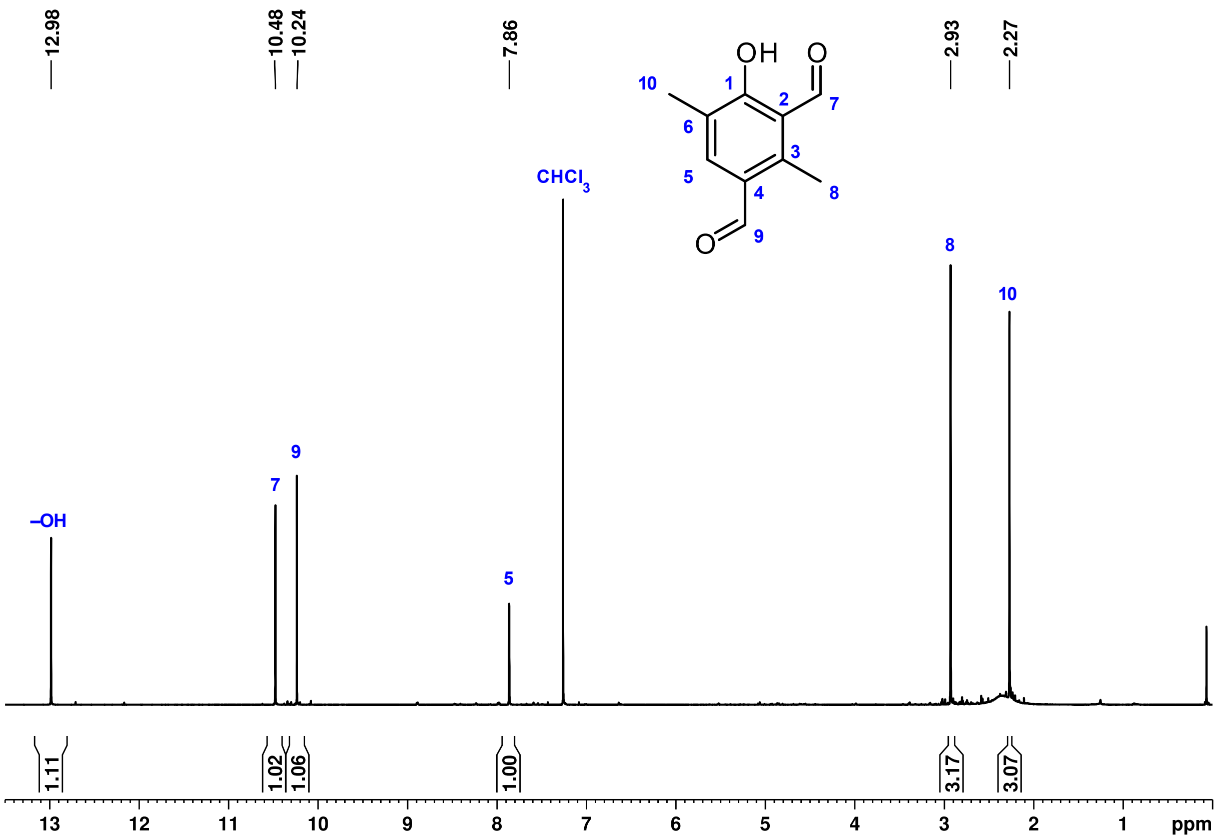

Figure S1. ^{1}H NMR spectrum of 4-hydroxy-2,5-dimethylisophthalaldehyde recorded in $CDCl_3$.

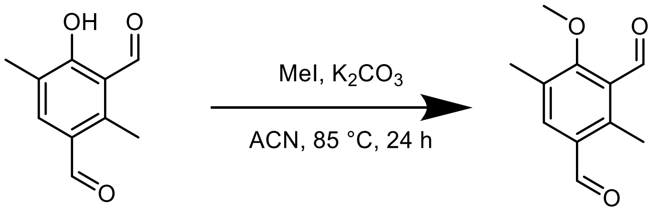

Procedure S2 – 4-Methoxy-2,5-dimethylisophthalaldehyde: In a 250 mL round-bottom flask, 4-hydroxy-2,5-dimethylisophthalaldehyde (1.43 g, 8.03, 1.00 eq.) was dissolved in 100 mL dry acetonitrile under inert conditions. Methyl iodide (0.75 mL, 1.50 eq.) was subsequently added in conjunction with anhydrous potassium carbonate (1.38 g, 1.25 eq.). The resulting suspension was left to stir at 85 °C for 24 h until consumption of the starting materials. After cooling to room temperature, 150 mL 0.1 M HCl and 250 mL of ethyl acetate were added, the phases separated, and the aqueous phase washed twice with 50 mL ethyl acetate. The combined organic phases were washed with brine, dried over $MgSO_4$ and the solvent was removed. The crude product was purified by flash chromatography (cyclohexane : ethyl acetate 85:15 v/v). The desired product was obtained as colorless crystals (0.78 g, 51% yield).

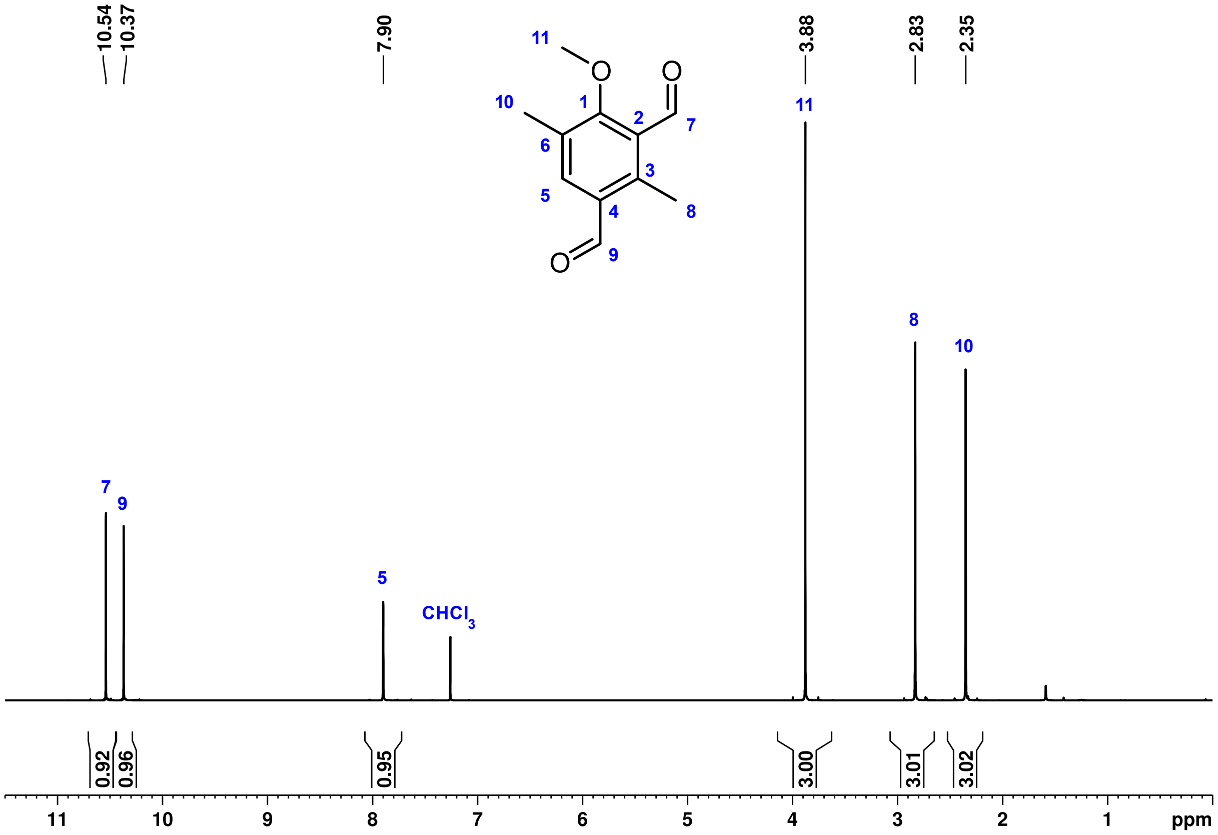

Figure S2. ^{1}H NMR spectrum of 4-methoxy-2,5-dimethylisophthalaldehyde recorded in $CDCl_3$.

Microsphere synthesis and E2-elimination

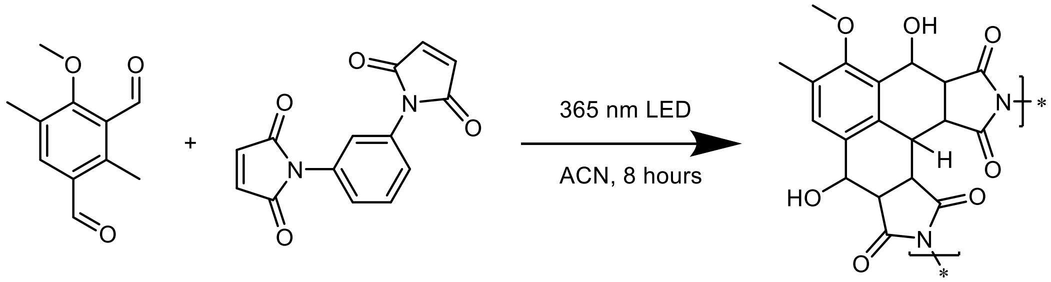

Procedure S3 – Microsphere synthesis: 4-Methoxy-2,5-dimethylisophthalaldehyde (42.5 mg, 0.22 mmol, 1 eq.) and N,N'-(1,3-Phenylene)dimaleimide (59.0 mg, 0.22 mmol, 1 eq.) were dissolved separately in 11 mL of acetonitrile (ACN) (20 mmol L^{-1}). The solutions were filtered through 2.5 µm PTFE syringe filter and 10 mL of each solution were added in a 20 mL clear crimp vial. The mixture was then bubbled with nitrogen for 5 min. The vial was subsequently placed on a bottle roller (10 rotations/min) and irradiated with a 365 nm 3W LED. After 8 hours, the turbid solution was centrifuged (9000 rpm, 10 min), the supernatant was removed, and the microspheres were washed with THF twice. The microspheres were redispersed in ACN and characterized by SEM (54.6 mg yield).

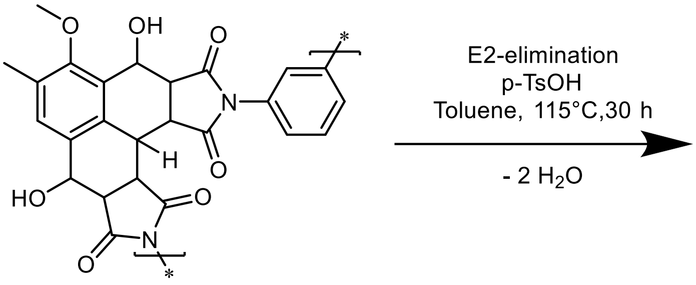

Procedure S4 – E2-elimination of the microspheres: The procedure is based on a previous study and utilized without further modification.[2] Briefly, the microspheres (25.1 mg) were dispersed in 34 mL of toluene in a 100 mL round-bottom pressure flask (V = 100 mL, p_{max} = 120 psi), along with 6.9 mg of p-toluenesulfonic acid monohydrate. The flask was sealed and sonicated for 15 min, after which the suspension was degassed with nitrogen for 10 min and left to stir at 125 °C for 72 h. After cooling at room temperature, the mixture was centrifuged (3000 rpm) and the pellet washed two times with DMSO and two times with toluene. The E2-eliminated microspheres were dried for 24 hours under high vacuum (8.4 mg yield).

FTIR spectra

FTIR (on Pt): v = 3500 (m), 3380 (m), 3068 (m), 2948 (s), 2933 (s), 2873 (w), 2840 (w), 2728 (w), 2258 (w), 2210 (w), 1935 (w), 1768 (s), 1714 (vs), 1600 (s), 1493 (s), 1452 (s), 1368 (s), 1282 (m), 1232 (s), 1168 (s), 1110 (m), 1068 (m), 1001 (s) 955 (w), 900 (m), 879 (m), 840 (m), 784 (s), 743 (m), 686 (s), 621 cm^{-1} (s)

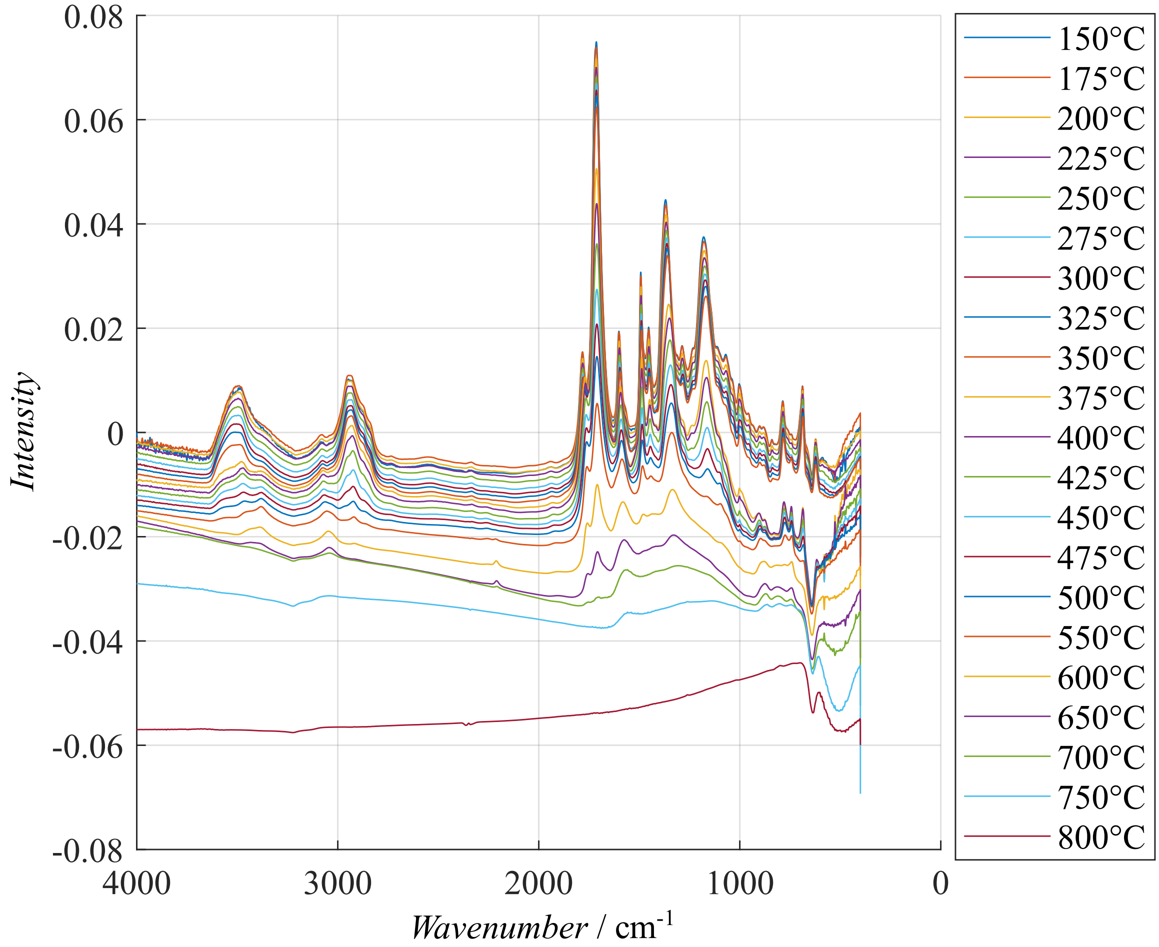

Figure S3. FTIR spectra of the pristine microspheres recorded at temperatures spanning 150 to 800°C, plotted for the full spectral range recorded. At temperatures above 350°C a screen is used (Nicolet screen A) to avoid saturation of the MTC detector, causing a discontinuity in the base line when transitioning from 350 to 375°C.

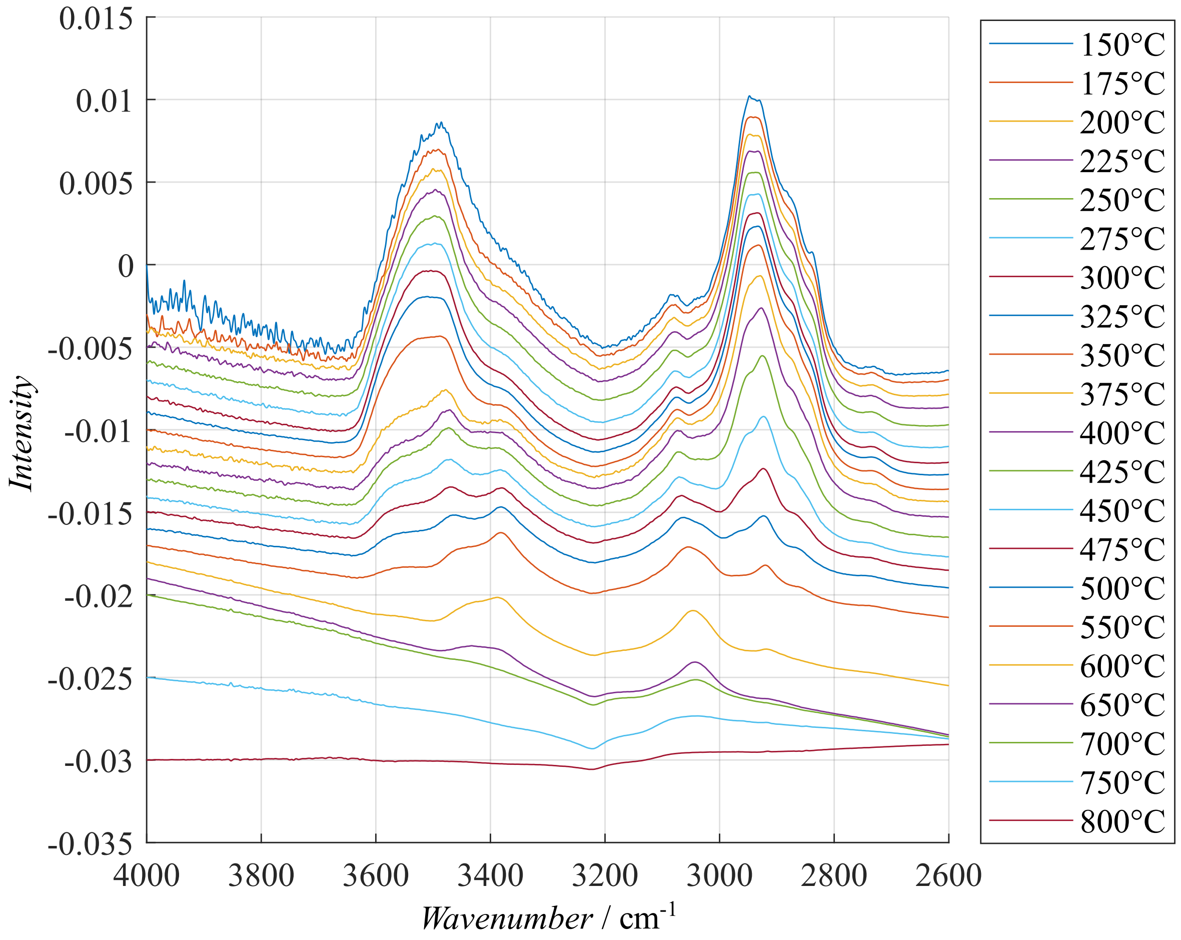

Figure S4. FTIR spectra of the pristine microspheres recorded at temperatures spanning 150 to 800°C, plotted for the 2600 cm^{-1} to 4000 cm^{-1} range. Temperature increases for spectra plotted at lower vertical axis values.

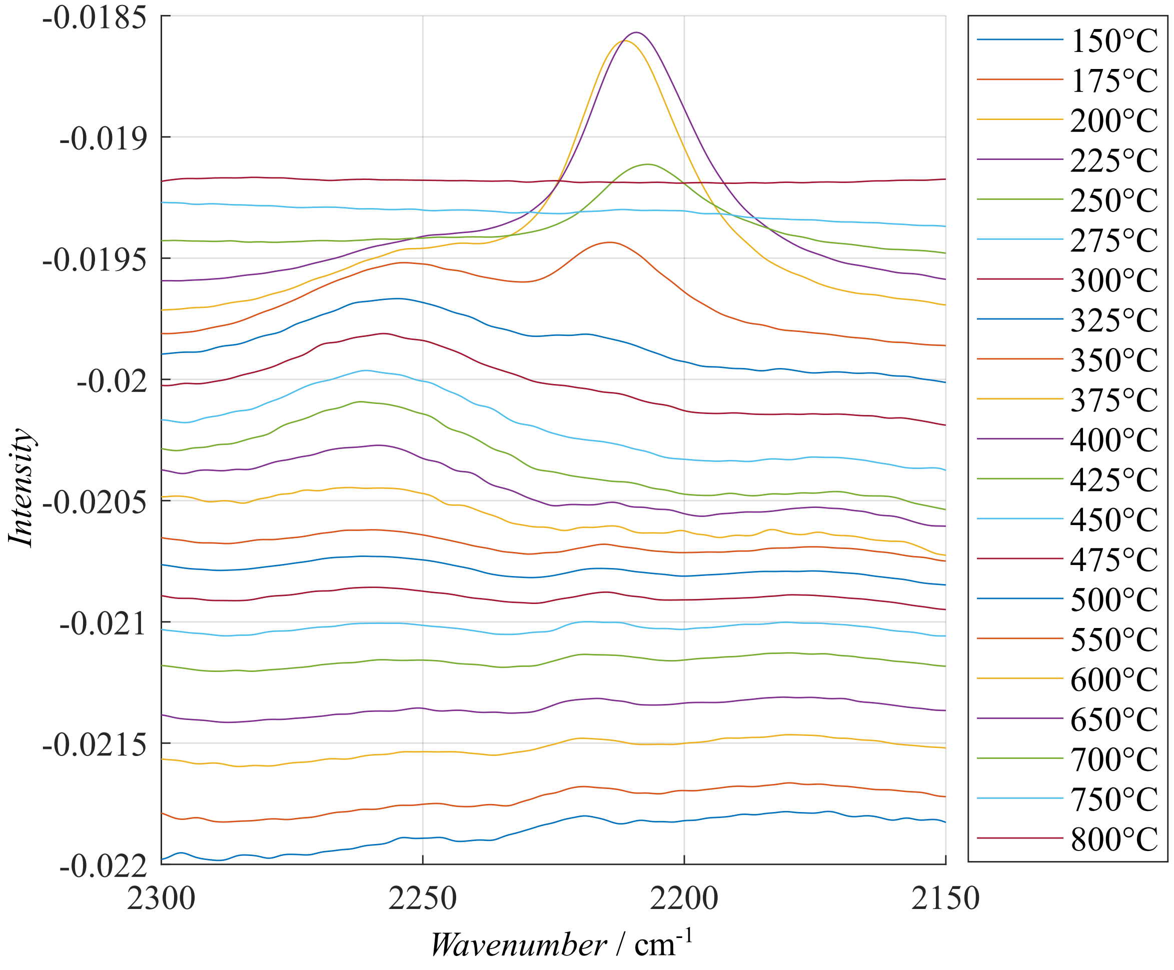

Figure S5. FTIR spectra of the pristine microspheres recorded at temperatures spanning 150 to 800°C, plotted for the 2150 cm^{-1} to 2300 cm^{-1} range. Temperature increases for spectra plotted at higher vertical axis values.

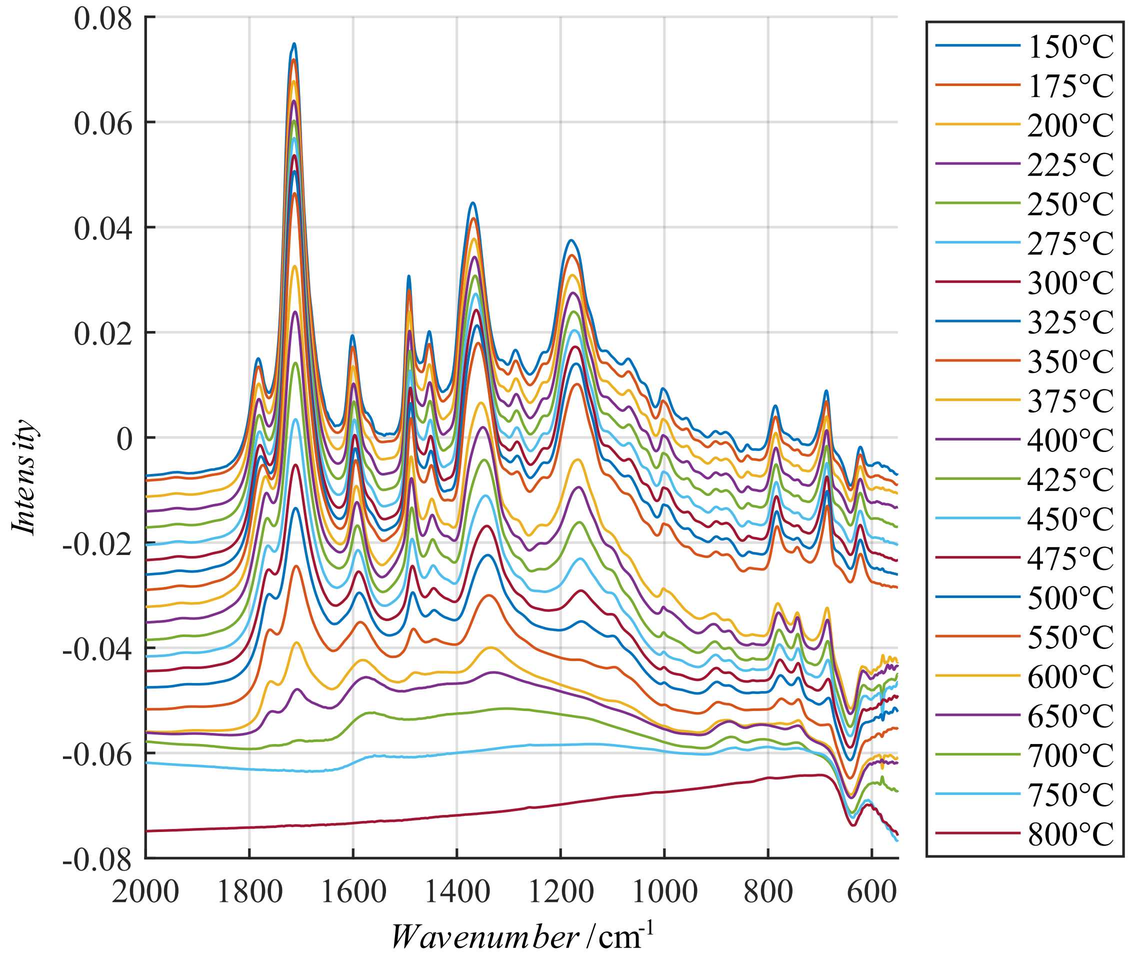

Figure S6. FTIR spectra of the pristine microspheres recorded at temperatures spanning 150 to 800°C, plotted for the 2000 cm^{-1} to 560 cm^{-1} range. Temperature increases for spectra plotted at lower vertical axis values.

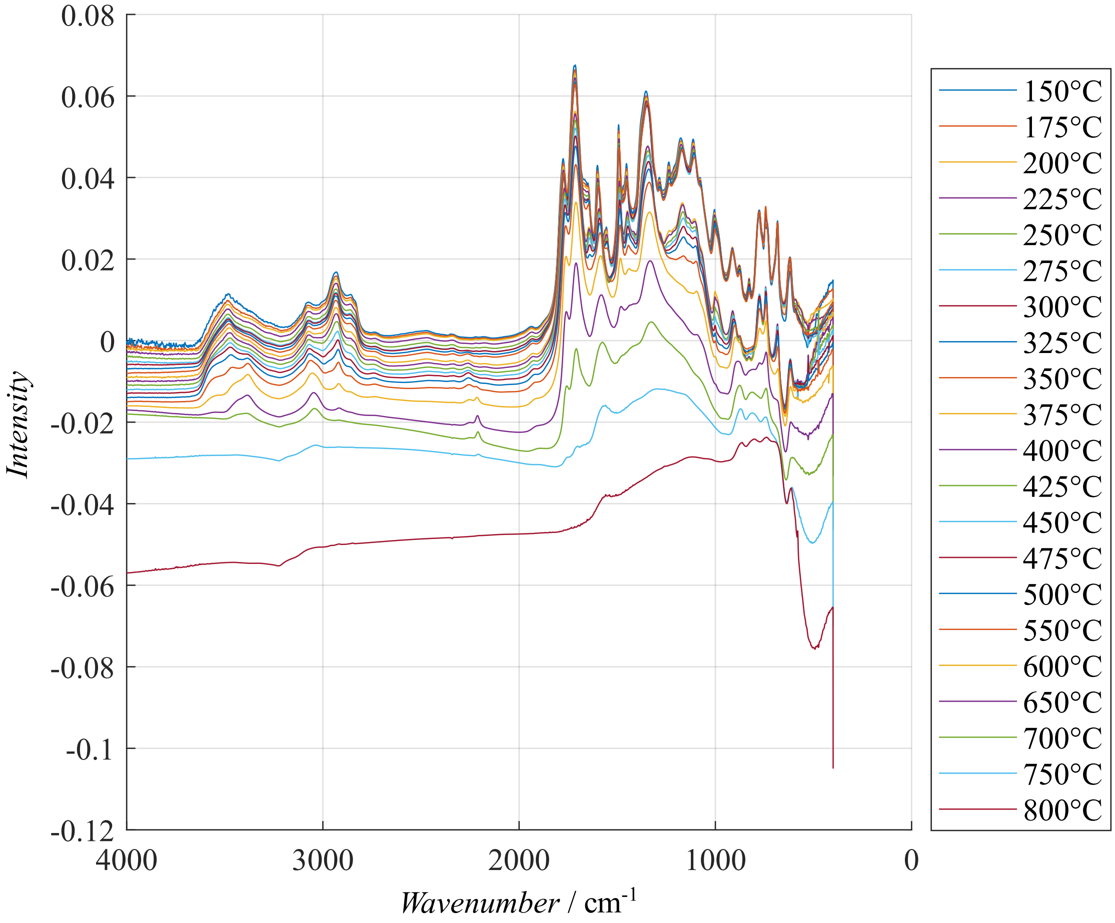

Figure S7. FTIR spectra of the hydroxyl eliminated microspheres recorded at temperatures spanning 150 to 800°C, plotted for the full spectral range recorded. At temperatures above 350°C a screen is used (Nicolet screen A) to avoid saturation of the MTC detector, causing a discontinuity in the base line when transitioning from 350 to 375°C.

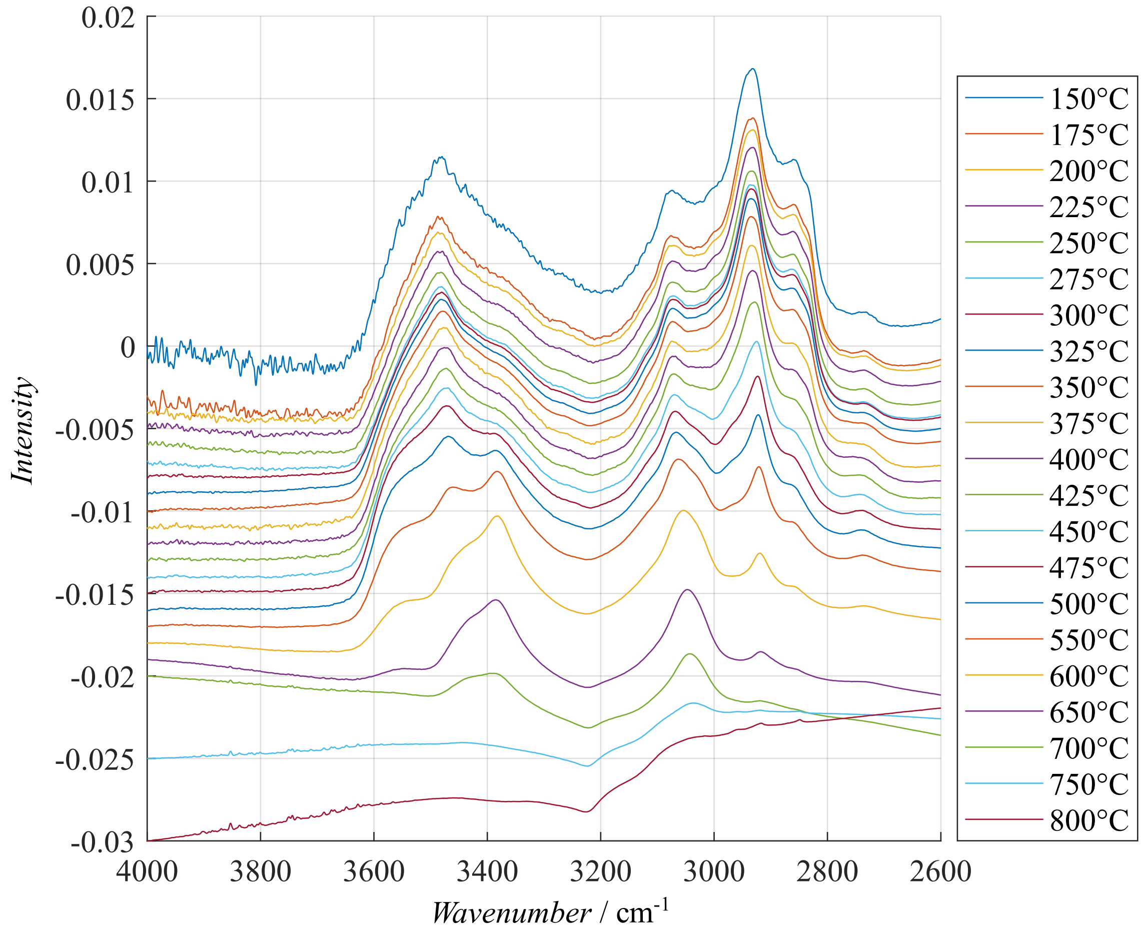

Figure S8. FTIR spectra of the hydroxyl eliminated microspheres recorded at temperatures spanning 150 to 800°C, plotted for the 2600 cm^{-1} to 4000 cm^{-1} range. Temperature increases for spectra plotted at lower vertical axis values.

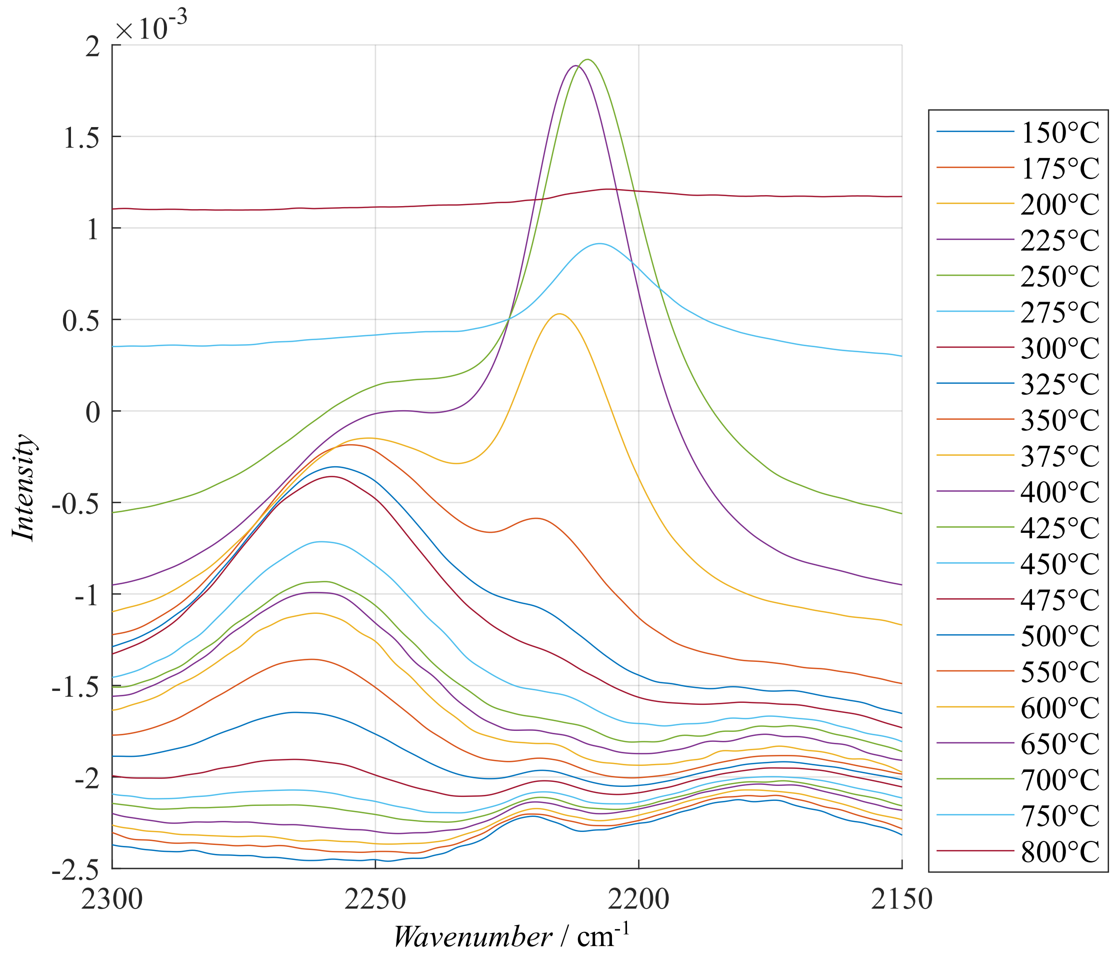

Figure S9. FTIR spectra of the hydroxyl eliminated microspheres recorded at temperatures spanning 150 to 800°C, plotted for the 2150 cm^{-1} to 2300 cm^{-1} range. Temperature increases for spectra plotted at higher vertical axis values.

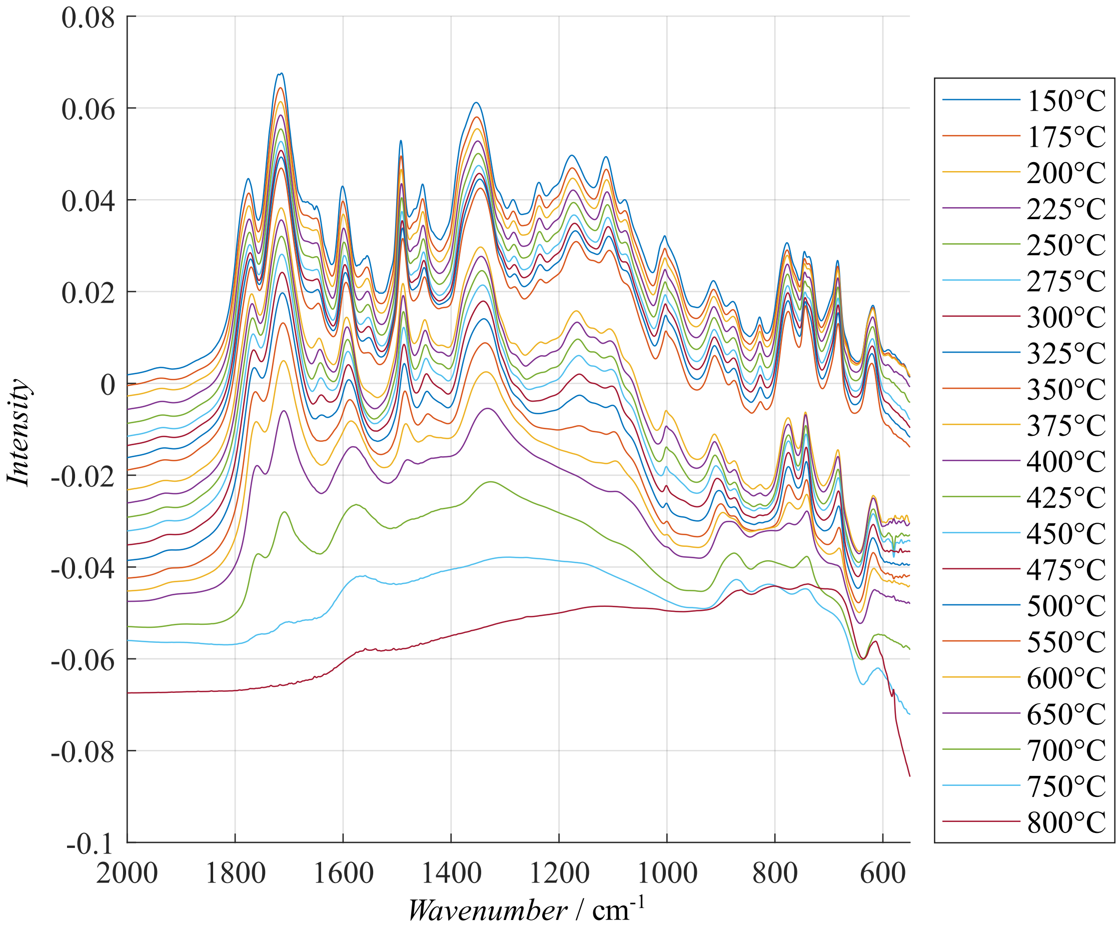

Figure S10. FTIR spectra of the hydroxyl eliminated microspheres recorded at temperatures spanning 150 to 800°C, plotted for the 2000 cm^{-1} to 560 cm^{-1} range. Temperature increases for spectra plotted at lower vertical axis values.

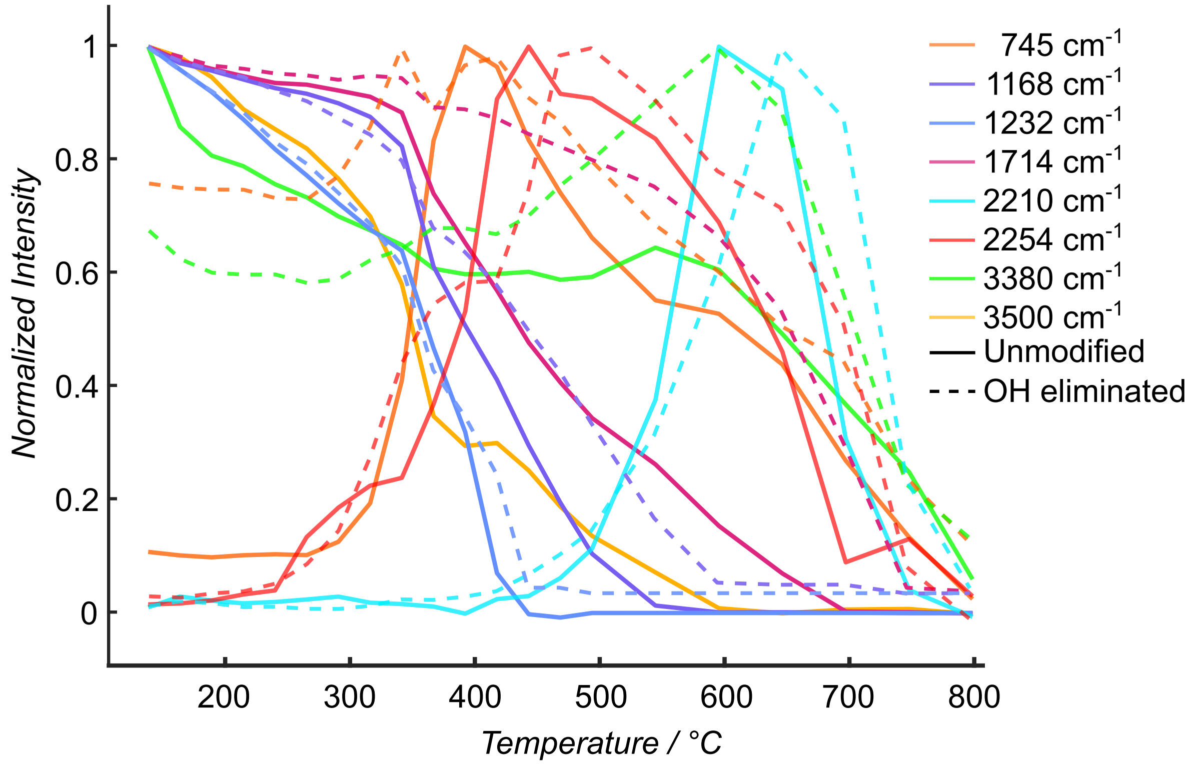

Figure S11. Normalized intensities for selected vibrations investigated with emission FTIR recorded at temperatures spanning 150 to 800°C. 745 cm^{-1} is attributed to *cis*-vinylene, 1168 cm^{-1} is attributed to the succinimide ring, 1232 cm^{-1} is attributed to the methoxy group, 1714 cm^{-1} is attributed to the ketones of the succinimide ring, 2210 cm^{-1} is attributed to a nitrile, 2254 cm^{-1} is attributed to an isocyanate, 3380 cm^{-1} is attributed to an aromatic alcohol, 3500 cm^{-1} is attributed to a secondary alcohol.[3] All intensities are evaluated by the local intensity of the selected vibration as compared to a linear fit as illustrated in Figure S12.

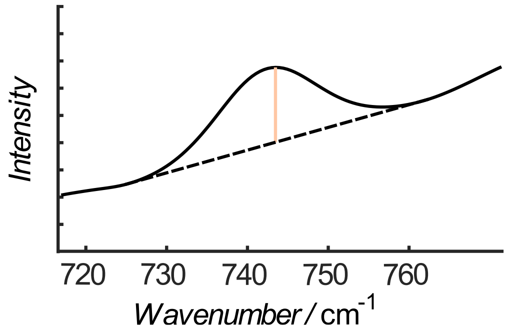

Figure S12. FTIR local intensity, measured as the peak intensity compared to a fitted linear background, is used to monitor the evolution of selected motifs. An example of how this local intensity is evaluated is given as an orange vertical line for the 745 cm^{-1} vibration.

Table S1. Measurement setting for recorded FT-IR spectra

Temperature [°C]	*Number of scans*	*Screen**
150-175	1024	-
200-300	512	-
325-350	256	-
375-400	256	A
425-800	128	A

*A screen is used to reduce the intensity of the IR light emitted by the sample to prevent saturation of the MTC detector at 375°C and higher temperatures.

Thermogravimetric measurements

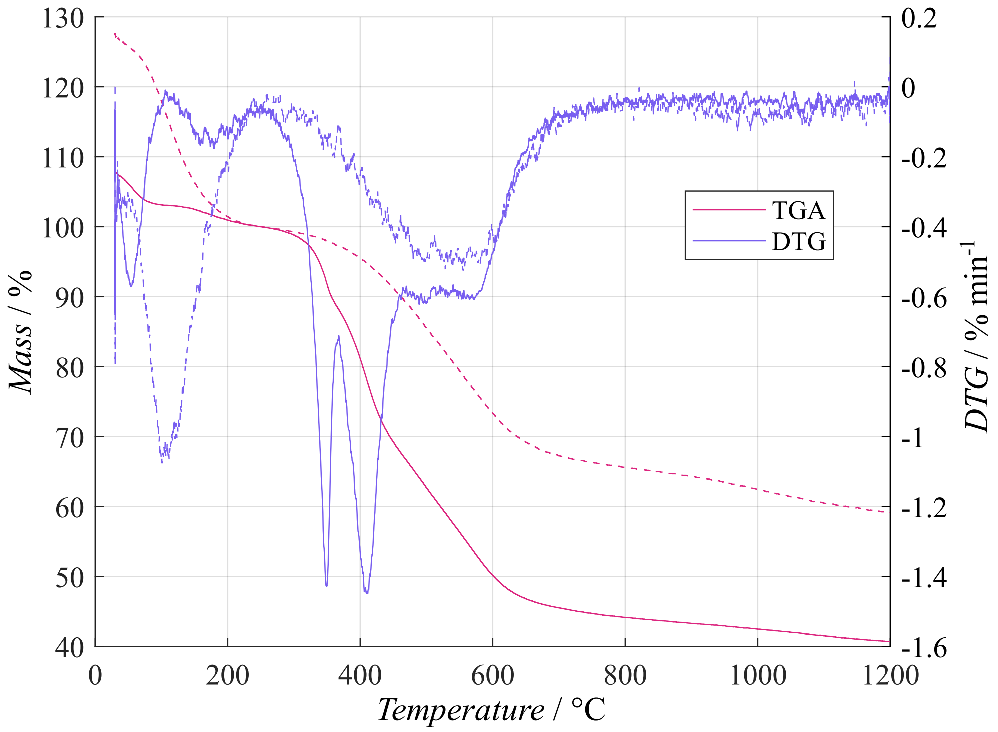

Figure S13. Thermogravimetric analysis with mass recorded at 5°C °C min^{-1} (TGA) and the differential of the thermogram (DTG) for pristine (solid lines) and hydroxyl eliminated (stippled lines) microspheres. The mass loss is corrected for solvent evaporation of toluene and ethanol persisting from the synthesis and washing steps, with ethanol being the first and toluene the

second mass loss below 250°C . The residual mass at 800°C is 44.2% and at 1200°C 40.7% for the pristine microspheres, and 65.5% at 800°C and 59.1% at 1200°C for the eliminated microspheres**.**

SEM images of microspheres

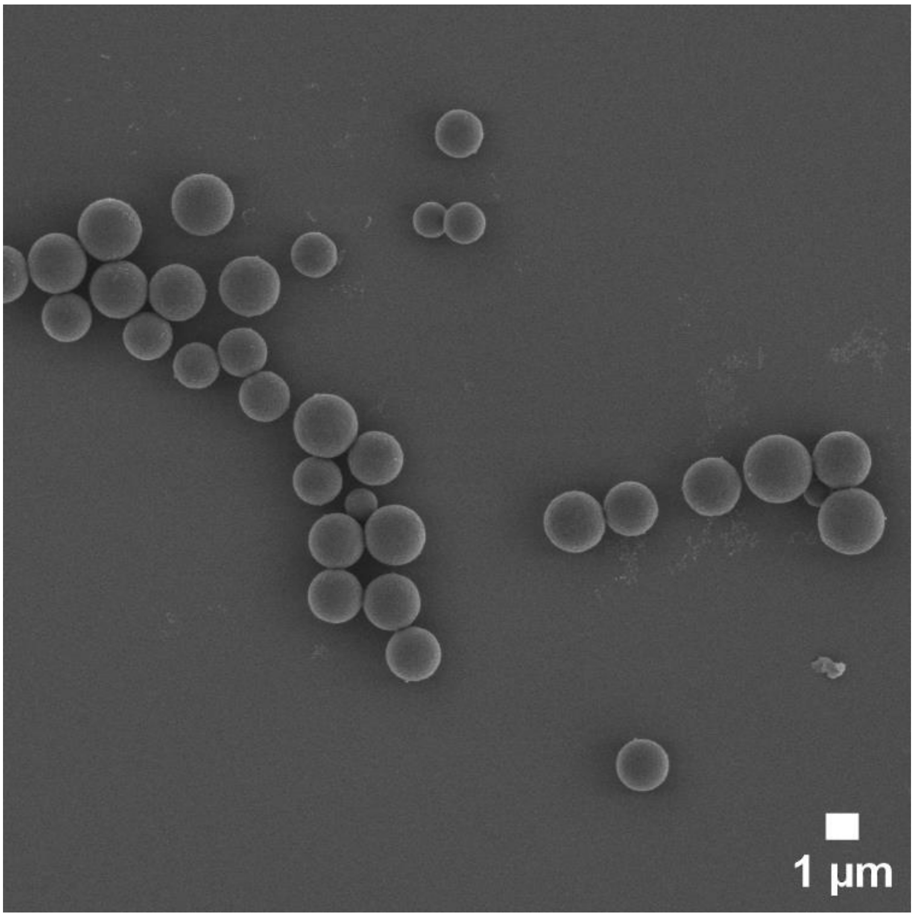

Figure S14. Example of **an** SEM image used to determine the average size of the pristine microspheres prior to carbonization. The microspheres were suspended in methanol, pipetted onto a Si wafer and sputtered with Pt before imaging.

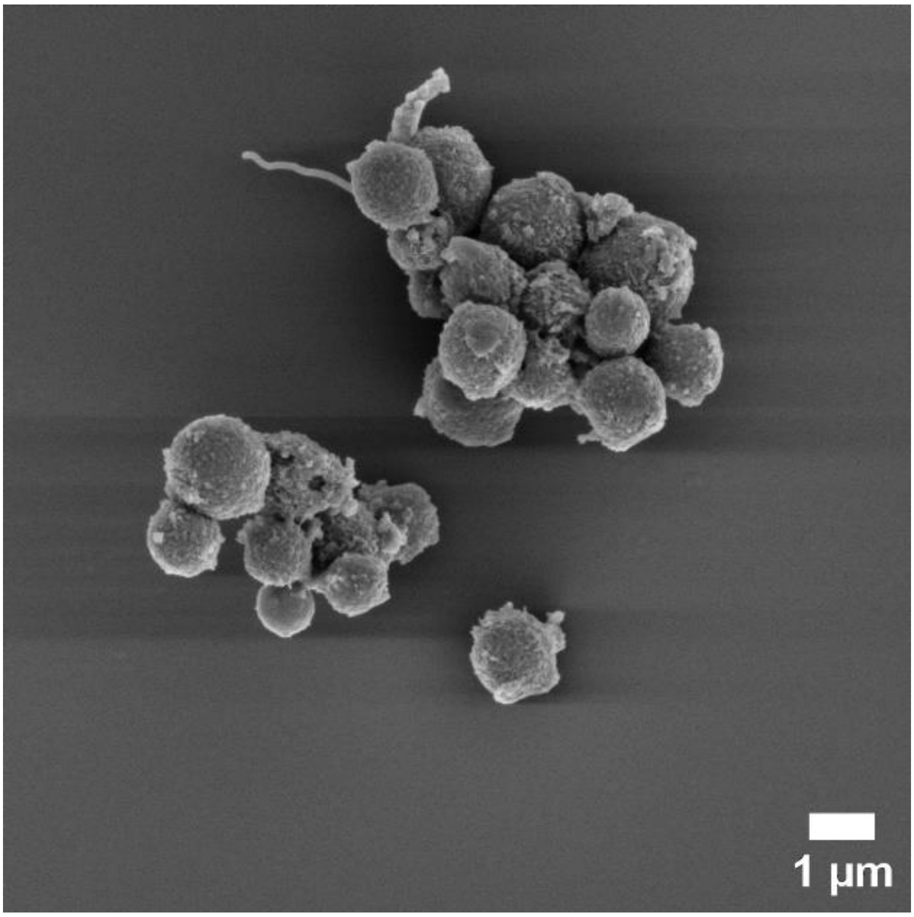

Figure S15. Example of an SEM image used to determine the average size of the hydroxyl eliminated microspheres after carbonization at 1200°C. The microspheres were suspended in methanol, pipetted onto a Si wafer and sputtered with Pt before imaging.

References

[1] F. Feist, S. L. Walden, J. Alves, S. V. Kunz, A. S. Micallef, A. J. Brock, J. C. McMurtrie, T. Weil, J. P. Blinco, C. Barner-Kowollik, *Angew Chem Int Ed* **2021**, *60*, 10402.

[2] J. A. Kammerer, F. Feist, D. Ryklin, A. Sarkar, C. Barner-Kowollik, R. R. Schröder, *Advanced Materials* **2023**, *35*, 2211074.

[3] G. Socrates, *Infrared and Raman Characteristic Group Frequencies: Tables and Charts*, Wiley, Chichester, **2010**.
